# Supplementary material for: In vivo antimicrobial activity of engineered mesoporous silica nanoparticles targeting intracellular mycobacteria
Source: Nat Commun. 2025 Aug 11;16:7388. doi: 10.1038/s41467-025-62623-y (PMC12339949; doi:10.1038/s41467-025-62623-y)
Supplement: Supplementary file 1 — Supplementary information [file 41467_2025_62623_MOESM1_ESM.pdf]

Supplementary information

In vivo antimicrobial activity of engineered mesoporous silica nanoparticles targeting intracellular mycobacteria

John Jairo Aguilera-Correa[1,2]*, Yara Tasrini[1]*, Miguel Gisbert-Garzarán[3] *, Aude Boulay[1], Tamara Carvalho[1], Fabien P. Blanchet[1,4], María Vallet-Regí[3] and Laurent Kremer[1,4,#]

[1]Centre National de la Recherche Scientifique UMR 9004, Institut de Recherche en Infectiologie de Montpellier (IRIM), Université de Montpellier, 1919 route de Mende, 34293, Montpellier, France.

[2]CIBERINFEC-CIBER de Enfermedades Infecciosas. 28029 Madrid, Spain.

[3]Departamento de Química en Ciencias Farmacéuticas, Universidad Complutense de Madrid, Instituto de Investigación Sanitaria Hospital 12 de Octubre i + 12, Plaza Ramón y Cajal s/n, 28040 Madrid, Spain

[4]INSERM, IRIM, 34293 Montpellier, France.

*These authors have contributed equally.

[#]To whom correspondence should be addressed: Tel: (+33) 4 34 35 94 47; E-mail: laurent.kremer@irim.cnrs.fr

Running title: Functionalized MSN target intracellular *M. marinum*

Keywords: *Mycobacterium marinum,* mesoporous silica nanoparticles, local therapy, drug delivery, macrophage, zebrafish, infection.

Supplementary Methods

Clarithromycin (CLR) loading and release. To produce CLR-loaded nanoparticles, 4-6 mg of MSN-AVA-TPP were dispersed in 1 mL of a 4 mg/mL solution of CLR (Sigma Aldrich, USA) in methanol (Honeywell, USA). The solution was stirred at 1,400 rpm at 4 °C overnight. The antibiotic-loaded nanoparticles were centrifuged, and the supernatant was removed. The CLR-loaded nanoparticles were dried at room temperature for at least 5 h under a chemical hood. To determine the kinetics of drug release from the nanoparticles, 2 mg of the CLR-loaded MSN-AVA-TPP were suspended in 0.5 mL of Phosphate Buffered Saline (PBS, Sigma Aldrich, USA) and placed into the upper chamber of a 12-well plate Transwell® (0.4 µm-diameter pore, Corning, USA). The lower chamber was filled with 1.5 mL of PBS and the plate incubated at 37 °C (n=3). Periodically, the whole volume from the lower chamber was collected and 1.5 mL of fresh PBS was subsequently added. The CLR concentration was determined in a LC-MS8030 Shimadzu (LC-ESI-QQQ-MS) mass spectrometer. A Poroshell 120 Phenyl-Hexyl column (2.1 mm x 50 mm, 2.7 µm) was employed. The samples were initially diluted in methanol prior to the analysis (5 µL sample + 995 µL methanol). The injection volume was set at 10 µL. The following gradient mode was employed: 0% Phase B - 4 min 95% Phase B – 6 min 95% Phase B – 6.5 min 0% Phase B. Phase A was 0.1% formic acid + 5 mM ammonium acetate in water LC-MS grade and phase B was methanol. The flow was set at 0.3 mL/min and the run time was 8 min.

Minimal inhibitory concentration and minimal bactericidal concentration. Minimum inhibitory concentration (MIC) was determined using a previously described broth microdilution method [1] with one modification. The MIC is the minimum concentration required to inhibit the bacterial visible growth. In brief, concentrations of MSN-AVA-TPP ranging from 500 µg/mL to 1.973 µg/mL with a two-fold dilution were added to Cation-adjusted Mueller-Hinton broth (CaMHB) (Sigma Aldrich, USA) to a final volume of 100

μL/well. 100 μL of mScarlet-*Mfort* in CaMHB containing approximately 3×10^6 CFU/mL were added to a 96-well Clear Round Bottom TC-treated Cell Culture Microplate (Corning Inc., USA) followed by static incubation at 30 °C for at least 3 days. After incubation, the MIC was determined visually as the lowest concentration of nanoparticles where no mycoacterial growth was observed. The minimum bactericidal concentration (MBC) was determined using the flash microbiocide method [2]. The MBC is defined as the minimum concentration required to kill bacteria. Briefly, 20 μL of the corresponding well from the MIC 96-wells plate were mixed with 180 μL of Middlebrook 7H9 supplemented with 10% OADC and 0.025% tyloxapol in a new 96-well plate, which was then incubated statically at 37 °C for 3 days. After incubation, the MBC was determined visually as the lowest concentration of nanoparticles where no mycobacterial growth was observed. The experiment was performed using four biological replicates.

Minimal biofilm inhibitory concentration and minimal biofilm eradication concentration. Minimal biofilm inhibitory concentration (MBIC) and minimal biofilm eradication concentration (MBEC) were determined using a previously described methodology [3]. The MBIC is the minimum concentration required to inhibit the visible growth of a bacterial biofilm. For the MBIC, a biofilm was formed by inoculating 100 μL of CaMHB containing 3×10^6 CFU/mL of mScarlet-*Mfort* on the bottom of the wells of a Nunc™ MicroWell™ 96-well, non-treated, flat-bottom plate (Thermo Fisher Scientific, United States) and the plate was statically incubated at 30 °C for 24 h prior to removal of the supernatant. Afterwards, each well was filled with 200 μL of CaMHB containing different concentrations of the corresponding nanoparticles, ranging from 500 μg/mL to 1.973 μg/mL with a two-fold dilution and the plate was statically incubated at 30 °C for 3 days. The MBIC was determined visually as the lowest concentration of nanoparticles where no planktonic mycobacterial growth from the biofilm was observed. For the MBEC, the bottom of each well was scrapped with a 100 μL tip to physically detach the biofilm from the bottom surface of each well. Then, 20 μL of each well were transferred to a new

well containing 180 µL of Middlebrook 7H9 supplemented with 10% OADC and 0.025% tyloxapol and the plate was statically incubated at 37 °C for 3 days. The MBEC was determined visually as the lowest concentration of nanoparticles where no mycobacterial growth was observed. The experiment was performed using four biological replicates.

***M. fortuitum* zebrafish infection and treatment.** AB (wild type) zebrafish embryos were infected with mScarlet-*Mfort* as previously described [4]. Briefly, 30hpf embryos were injected intravenously with around 2500 CFU of mScarlet-*Mfort,* and treated intravenously with 20 ng of MSN-AVA-TPP@DOX at either 1dpi, or at 1dpi, 3dpi and 5dpi. Control embryos were included. The experiment was performed in 3 biological replicates with n=20 embryos per condition.

Supplementary Tables

Supplementary Table 1. Strains used in this study.

Strain	Description	Source
Mycobacterium marinum (*Mmar*)	M strain	[5]
mScarlet-*Mmar*	*Mmar* WT transformed with integrative plasmid pMV306-P_{left}**mScarlet*-Kan^R	This study
mWasabi-*Mmar*	*Mmar* WT transformed with integrative plasmid pMV306-P_{left}**mWasabi* Kan^R	This study
Mycobacterium abscessus (*Mab*)	CIP104536 (S), smooth variant	[6]
mScarlet-*Mab*	CIP104536 (S) transformed with integrative plasmid pMV306-P_{left}**mScarlet*-Kan^R	[7]
Mycobacterium avium (*Mav*)	MAC 101 ATCC 700898	ATCC
mScarlet-*Mav*	MAC 101 transformed with integrative plasmid pMV306-P_{left}**mScarlet*-Kan^R	This study
Mycobacterium smegmatis (*Msmeg*)	Strain mc^{2}155	[8]
tdTomato-*Msmeg*	*Msmeg* mc^{2}155 transformed with pTEC27 (tdTomato, Hyg^R)	This study
Mycobacterium fortuitum (*Mfort*)	ATCC 6841	ATCC
tdTomato-*Mfort*	*Mfort* transformed with pVV16-tdTomato (tdTomato, Kan^R)	This study
mScarlet-*Mfort*	*Mfort* WT transformed with integrative plasmid pMV306-P_{left}**mScarlet*-Kan^R	This study
Mycobacterium kansasii (*Mkan*)	ATCC 12478	ATCC
tdTomato-*Mkan*	*Mkan* transformed with pTEC27 (tdTomato, Hyg^R)	This study

Supplementary Table 2. Minimum inhibitory concentration (MIC) of *M. marinum* and *M. fortuitum* against clinically used drugs (Sensititre ™ RAPMYCO2).

[a]R, resistant; S, susceptible

		M. marinum mScarlet	*M. marinum* mWasabi	*M. fortuitum*	*M. fortuitum* mScarlet
**Antibiotic**	**Range (µg/mL)**	**MIC (µg/mL) (Phenotype)[a]**	**MIC (µg/mL) (Phenotype)**	**MIC (µg/mL) (Phenotype)**	**MIC (µg/mL) (Phenotype)**
Trimethoprim/Sulfamethoxazole	0.25/4.75-8/152	4/76 (R)	4/76 (R)	>8/152 (R)	>8/152 (R)
Linezolid	1-32	2 (S)	2 (S)	4 (S)	4 (S)
Ciprofloxacin	0.12/4	1(S)	1(S)	0.5 (S)	0.5 (S)
Imipenem	2-64	4 (R)	4 (R)	64 (S)	64 (S)
Moxifloxacin	0.25-8	1(S)	1(S)	0.5 (S)	0.5 (S)
Cefepime	1-32	>32 (R)	>32 (R)	>32 (R)	>32 (R)
Cefoxitin	4-128	>128 (R)	>128 (R)	64 (S)	64 (S)
Amoxicillin/Clavulanic acid (2:1 ratio)	2/1-64/32	64/32 (R)	64/32 (R)	32/16 (S)	32/16 (S)
Amikacin	1-64	2 (S)	2 (S)	2 (S)	2 (S)
Ceftriaxone	4-64	>64 (R)	>64 (R)	>64 (R)	>64 (R)
Doxycycline	0.12-16	1(S)	1(S)	0.5 (S)	0.5 (S)
Minocycline	1-8	>8 (R)	>8 (R)	1 (S)	1 (S)
Tigecycline	0.015-4	1(S)	1(S)	0.25 (S)	0.25 (S)
Tobramycin	1-16	16 (R)	16 (R)	16 (S)	16 (S)
Clarithromycin	0.06-16	4 (S)	4 (S)	16 (S)	16 (S)

Supplementary Table 3. Minimum inhibitory concentration (MIC), Minimum bactericidal concentration (MBC), Minimum biofilm inhibitory concentration (MBIC) and Minimum biofilm eradication concentration (MBEC) of MSN-AVA-TPP@DOX and MSN-AVA-TPP@CLR against *M. marinum* expressing mScarlet.

		MSN-AVA-TPP@DOX (µg/mL)	MSN-AVA-TPP@CLR (µg/mL)
M. marinum	MIC	125	15.6
	MBC	250	31.2
	MBIC	250	15.6
	MBEC	500	31.2

Supplementary Table 4. Minimum inhibitory concentration (MIC), Minimum bactericidal concentration (MBC), Minimum biofilm inhibitory concentration (MBIC) and Minimum biofilm eradication concentration (MBEC) of MSN-AVA-TPP@DOX against *M. fortuitum* expressing mScarlet.

	**MSN-AVA-TPP@DOX (µg/mL)**
**MIC**	15.6
**MBC**	62.5
**MBIC**	31.2
**MBEC**	250

Supplementary Figures

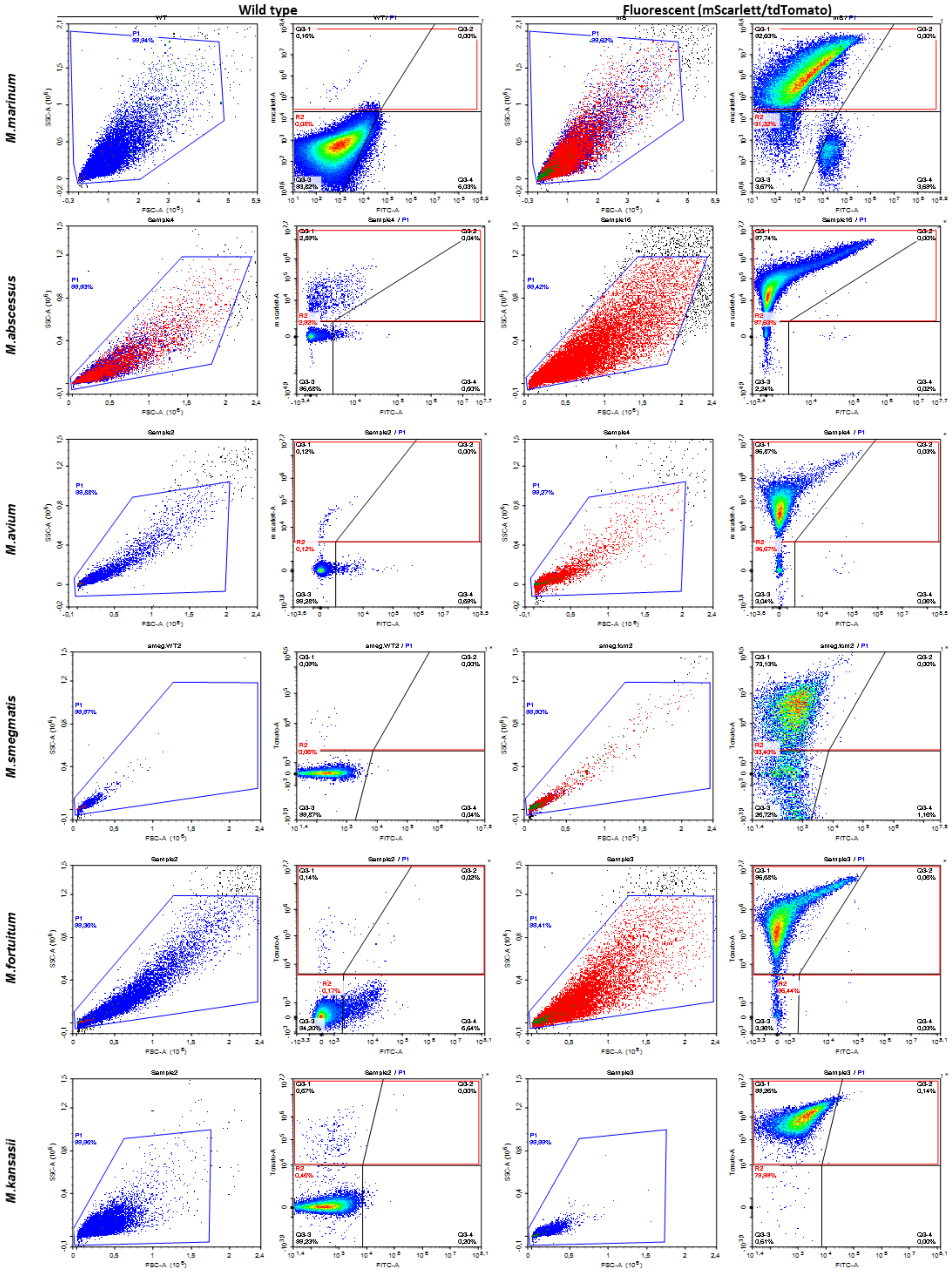

Supplementary Fig 1. Gating strategy for the cytometry analysis of the nanoparticle affinity with the different mycobacterial species. The first two columns correspond to the gating of the WT strains, and the third and fourth columns to the gating of the fluorescent strains.

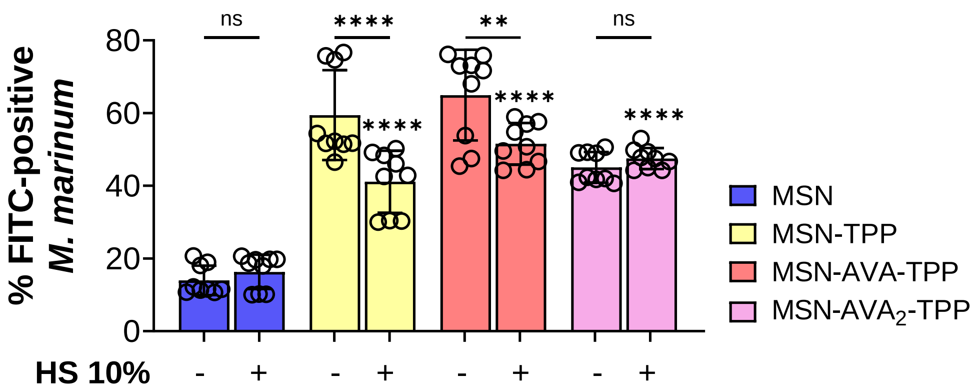

Supplementary Fig 2. Percentages of FITC-positive *Mmar* incubated with 200 µg/mL of each functionalized nanosystem with and without 10% human serum (HS) using flow cytometry. Histograms were generated on the mycobacterial population expressing mScarlet. Three biological replicates and 3 technical replicates were performed, with at least 1 million events measured per technical replicate, and are plotted as mean ± SD per biological replicate (n=9). Comparisons were done using ordinary one-way ANOVA with Šídák's multiple comparisons test. Asterisks above each bar correspond to comparisons to MSN. Adjusted *p*-values (from left to right): ****p<0.0001, ns p=0.9997, **p=0.0540, ns p=0.9995.

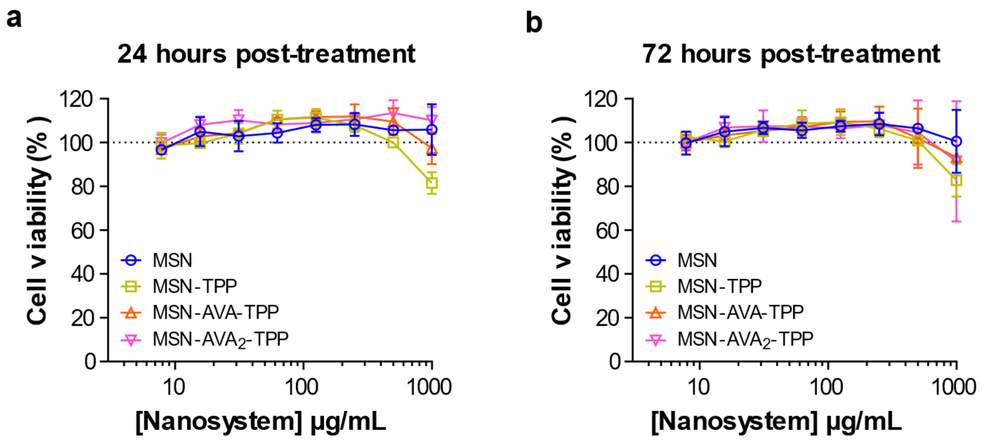

Supplementary Fig 3. Cell viability (using a resazurin assays) of THP-1 cells treated with different concentrations of each nanosystem **(a)** at 24 h and **(b)** at 72 h. Four biological replicates with three technical replicates each) were performed. Data are shown as mean ± SD of biological replicates (n=4).

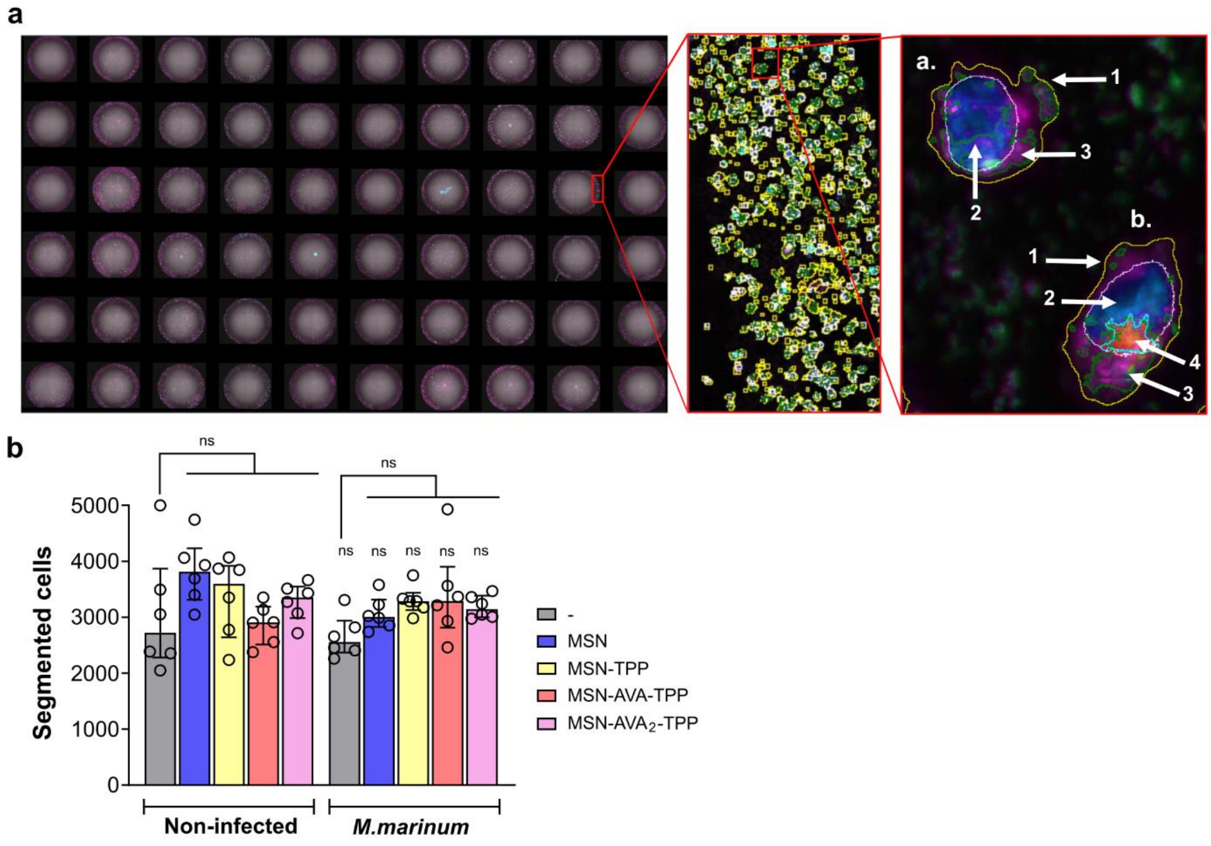

Supplementary Fig 4. Schematic view of the segmentation used in this study. **(a)** Image of the full plate (left panel) with a zoom inside the red square as an example. The enlarged red square (middle panel) shows the different segmented cells. The right panel represents the zoom of the second panel showing: **a.** a non-infected cell; **b.** an infected cell. White arrows indicate segmented events as following: **1:** WGA segmentation (cell membrane); **2:** DAPI segmentation (Nucleus); **3:** FITC segmentation (nanoparticle); **4:** mScarlet segmentation (*M. marinum*). **(b)** Quantification of the number of segmented cells under the non-infected and *M. marinum*-infected conditions. 3 biological replicates and 3 technical replicates were performed. Data are shown as median and interquartile range. Comparisons were done using ordinary one-way ANOVA with Šídák's multiple comparisons test. Asterisks above each bar correspond to the comparison to the non-infected same condition. ns: non-significant.

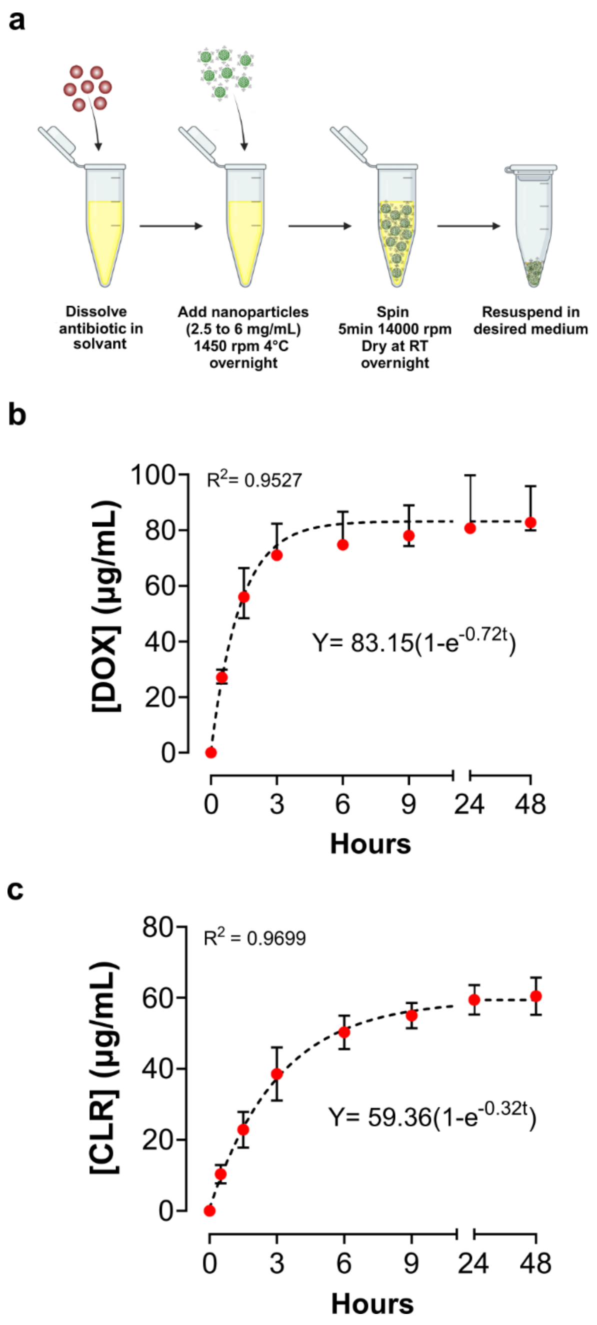

Supplementary Fig 5. (a) Schematic representation of the antibiotic-loading protocol. **(b)** Doxycycline (DOX) release from 3 mg/mL of MSN-AVA-TPP@DOX. Dots represent the median and the error bars indicate the interquartile range. **(c)** Clarithromycin (CLR) release from 3 mg/mL of MSN-AVA-TPP@CLR. Dots represent the mean ± SD. Created in BioRender. Kremer, L. (2025) https://BioRender.com/pgdahvf.

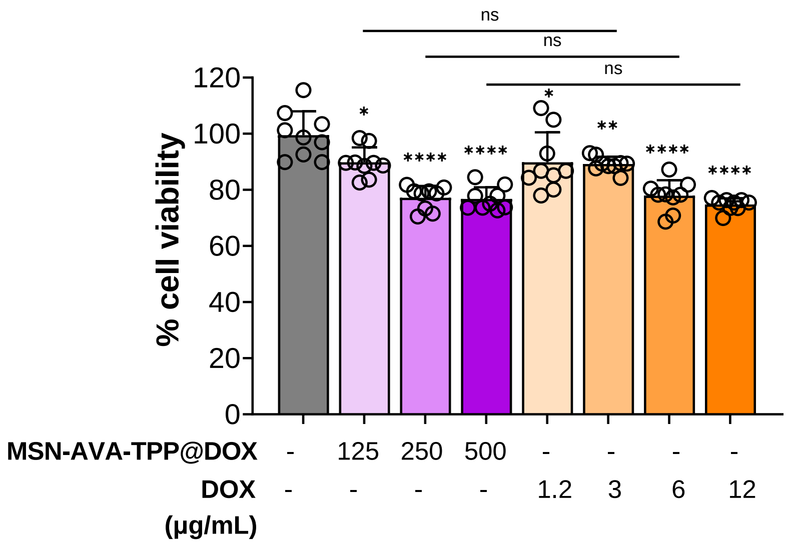

Supplementary Fig 6. Cell viability (MTS assay) of THP-1 cells treated with MSN-AVA-TPP@DOX (125, 250 and 500 µg/mL) or free DOX (1.2, 3, 6 and 12 µg/mL). Data are shown as mean ± SD. Three biological replicates and 3 technical replicates were performed (n=9). Comparisons were done using ordinary one-way ANOVA with Šídák's multiple comparisons test. Asterisks above each data set correspond to comparisons to untreated condition (grey). Adjusted *p*-values (from left to right): *p=0.0115 (AVA-TPP@DOX 125), ****p<0.0001, *p=0.0115 (DOX 1.2), **p=0.0058 (DOX 3), ns p>0.9999 (AVA-TPP@DOX 125 *vs* DOX 3), ns (non-significant) p>0.9999 (AVA-TPP@DOX 250 *vs* DOX 6), ns p=0.9988 (AVA-TPP@DOX 500 *vs* DOX 12).

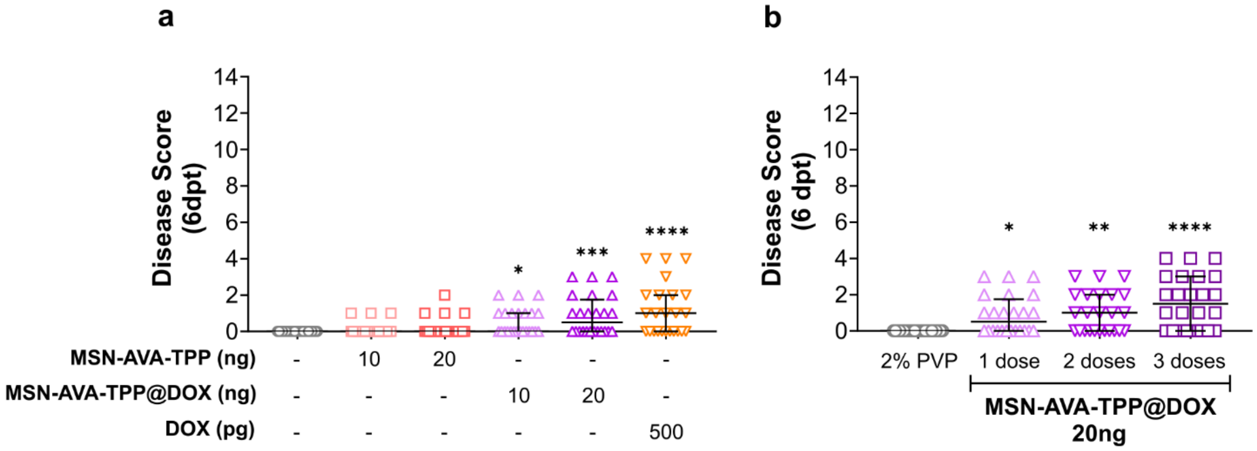

Supplementary Fig 7. Disease score of zebrafish embryos treated with (a) MSN-AVA-TPP, MSN-AVA-TPP@DOX and free DOX at 2 days post-fertilization or (b) with MSN-AVA-TPP@DOX at 2, 4- and 6-days post-fertilization. (a-b) The disease score was determined daily, and is only shown at the end of the experiment (6 days post-treatment) to assess toxicity. Comparisons were done using Kruskal-Wallis with Dunn's multiple comparisons test. Asterisks above each data set correspond to comparisons to untreated condition (grey). (a) Adjusted *p*-values: *p=0.0189, ***p=0.0008, ****p<0.0001; (b) Adjusted *p*-values: *p=0.0112, **p=0.0031, ****p<0.0001.

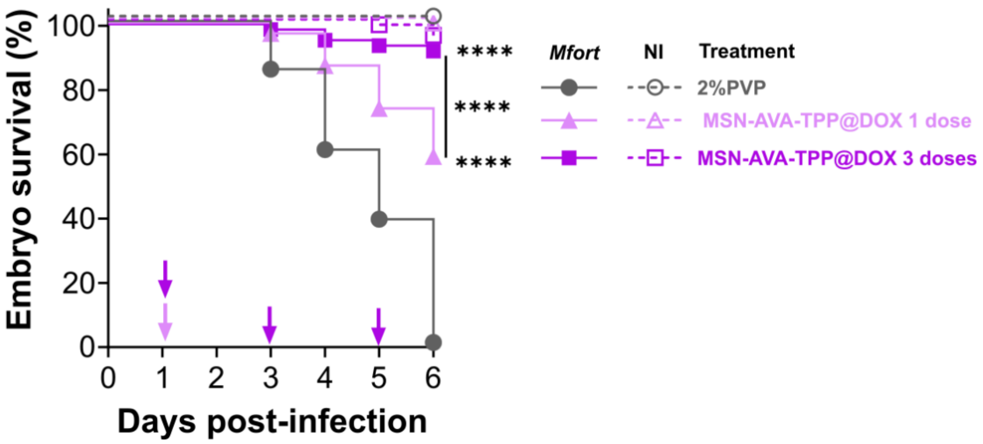

Supplementary Fig 8. Survival curve of infected embryos with *M. fortuitum* expressing mScarlet, treated with either one dose of MSN-AVA-TPP@DOX of 20 ng (at 1 dpi), or 3 doses (at 1 dpi, 3dpi and 5dpi). Arrows indicate the time of treatment. Dashed lines correspond to non-infected embryos with different treatment doses. Comparisons were done using a Log-rank (Mantel Cox) test. Asterisks next to survival curves correspond to comparisons with respective non-infected control. *p*-values: ****p<0.0001.

Supplementary References

1. Weinstein, M. P. Methods for dilution antimicrobial susceptibility tests for bacteria that grow aerobically. In Clinical and Laboratory Standards Institute, 11th Edition (2018).
2. Hernandes, C., Coppede, J. da S., Bertoni, B. W., França, S. de C. & Pereira, A. M. S. Flash microbiocide: A rapid and economic method for determination of MBC and MFC. *Am. J. Plant Sci.* **4**, 850–852 (2013).
3. Pettit, R. K. et al. Microplate alamar blue Aasay for *Staphylococcus epidermidis* biofilm susceptibility testing. Antimicrob Agents Chemother **49**, 2612–2617 (2005).
4. Johansen, M. D. & Kremer, L. CFTR depletion confers hypersusceptibility to *Mycobacterium fortuitum* in a zebrafish model. Front. Cell. Infect. Microbiol. **10**, 357 (2020).
5. Stinear, T. P. *et al.* Insights from the complete genome sequence of *Mycobacterium marinum* on the evolution of *Mycobacterium tuberculosis*. *Genome Res.* **18**, 729–741 (2008).
6. Ripoll, F. *et al*. Non mycobacterial virulence genes in the genome of the emerging pathogen *Mycobacterium abscessus*. *PLoS ONE* **4**, e5660 (2009).
7. Boudehen, Y.-M., Tasrini, Y., Aguilera-Correa, J. J., Alcaraz, M. & Kremer, L. Silencing essential gene expression in *Mycobacterium abscessus* during infection. *Microbiol. Spectr.* e0283623 (2023).
8. Snapper, S. B., Melton, R. E., Mustafa, S., Kieser, T. & Jacobs, W. R. Isolation and characterization of efficient plasmid transformation mutants of *Mycobacterium smegmatis. Mol. Microbiol.* **4**, 1911–1919 (1990).
