# Supplementary material for: In vivo antimicrobial activity of engineered mesoporous silica nanoparticles targeting intracellular mycobacteria
Source: Nat Commun. 2025 Aug 11;16:7388. doi: 10.1038/s41467-025-62623-y (PMC12339949; doi:10.1038/s41467-025-62623-y)
Supplement: Supplementary file 2 — Description of Additional Supplementary Files [file 41467_2025_62623_MOESM2_ESM.pdf]

Title: Supplementary Movie 1
Description: 3D representation of confocal z-stack image of THP-1 treated with MSN.

Title: Supplementary Movie 2
Description: 3D representation of confocal z-stack image of THP-1 treated with MSN-TPP.

Title: Supplementary Movie 3
Description: 3D representation of confocal z-stack image of THP-1 treated with MSN-AVA-TPP.

Title: Supplementary Movie 4
Description: 3D representation of confocal z-stack image of THP-1 treated with MSN-AVA2-TPP.

Title: Supplementary Movie 5
Description: 3D representation of confocal z-stack image of M. marinum-infected THP-1 treated with MSN.

Title: Supplementary Movie 6
Description: 3D representation of confocal z-stack image of M. marinum-infected THP-1 treated with MSN-TPP.

Title: Supplementary Movie 7
Description: 3D representation of confocal z-stack image of M. marinum-infected THP-1 treated with MSN-AVA-TPP.

Title: Supplementary Movie 8
Description: 3D representation of confocal z-stack image of M. marinum-infected THP-1 treated with MSN-AVA2-TPP.

Title: Supplementary Movie 9
Description: 3D representation of confocal z-stack image of M. marinum-infected THP-1 untreated.

Title: Supplementary Movie 10
Description: 3D representation of confocal z-stack image of M. marinum-infected THP-1 treated with MSN-AVA-TPP@DOX at 125 μg/mL.

Title: Supplementary Movie 11
Description: 3D representation of confocal z-stack image of M. marinum-infected THP-1 treated with MSN-AVA-TPP@DOX at 250 μg/mL.

Title: Supplementary Movie 12
Description: 3D representation of confocal z-stack image of M. marinum-infected THP-1 treated with MSN-AVA-TPP@DOX at 500 μg/mL. Supplementary Movie 13: 3D

representation of confocal z-stack image of M. marinum-infected zebrafish embryos treated with 20 ng of MSN-AVA-TPP@DOX after 24 h of treatment.

Title: Supplementary Movie 14
Description: 3D representation of confocal z-stack image of M. marinum-infected zebrafish embryos treated with one dose of 20 ng of MSN-AVA-TPP@DOX after 48 h of treatment.

Title: Supplementary Movie 15
Description: 3D representation of confocal z-stack image of M. marinum-infected zebrafish embryos treated with three doses of 20 ng of MSN-AVA-TPP@DOX 48 h after the last dose.
